# The transcription factors Ik-1 and MZF1 downregulate *IGF-IR* expression in NPM-ALK^+^ T-cell lymphoma

**DOI:** 10.1186/s12943-015-0324-2

**Published:** 2015-02-25

**Authors:** Deeksha Vishwamitra, Choladda V Curry, Serhan Alkan, Yao-Hua Song, Gary E Gallick, Ahmed O Kaseb, Ping Shi, Hesham M Amin

**Affiliations:** Department of Hematopathology, The University of Texas MD Anderson Cancer Center, 1515 Holcombe Boulevard, Houston, Texas USA; The University of Texas Graduate School of Biomedical Sciences, Houston, TX 77030 USA; Department of Pathology and Immunology, Baylor College of Medicine and Texas Children’s Hospital, Houston, Texas USA; Department of Pathology and Laboratory Medicine, Cedars-Sinai Medical Center, Los Angeles, California USA; Cyrus Tang Hematology Center, Jiangsu Institute of Hematology, First Affiliated Hospital, Collaborative Innovation Center of Hematology, Soochow University, Suzhou, China; Department of Genitourinary Medical Oncology, The University of Texas MD Anderson Cancer Center, Houston, Texas USA; Department of Gastrointestinal Medical Oncology, The University of Texas MD Anderson Cancer Center, Houston, Texas USA; State Key Laboratory of Bioreactor Engineering, East China University of Science and Technology, Shanghai, China

**Keywords:** IGF-IR, Ik-1, MZF1, NPM-ALK, T-cell lymphoma

## Abstract

**Background:**

The type I insulin-like growth factor receptor (IGF-IR) tyrosine kinase promotes the survival of an aggressive subtype of T-cell lymphoma by interacting with nucleophosmin-anaplastic lymphoma kinase (NPM-ALK) oncogenic protein. NPM-ALK^+^ T-cell lymphoma exhibits much higher levels of IGF-IR than normal human T lymphocytes. The mechanisms underlying increased expression of IGF-IR in this lymphoma are not known. We hypothesized that upregulation of IGF-IR could be attributed to previously unrecognized defects that inherently exist in the transcriptional machinery in NPM-ALK^+^ T-cell lymphoma.

**Methods and results:**

Screening studies showed substantially lower levels of the transcription factors Ikaros isoform 1 (Ik-1) and myeloid zinc finger 1 (MZF1) in NPM-ALK^+^ T-cell lymphoma cell lines and primary tumor tissues from patients than in human T lymphocytes. A luciferase assay supported that Ik-1 and MZF1 suppress *IGF-IR* gene promoter. Furthermore, ChIP assay showed that these transcription factors bind specific sites located within the *IGF-IR* gene promoter. Forced expression of Ik-1 or MZF1 in the lymphoma cells decreased IGF-IR mRNA and protein. This decrease was associated with downregulation of pIGF-IR, and the phosphorylation of its interacting proteins IRS-1, AKT, and NPM-ALK. In addition, overexpression of Ik-1 and MZF1 decreased the viability, proliferation, migration, and anchorage-independent colony formation of the lymphoma cells.

**Conclusions:**

Our results provide novel evidence that the aberrant decreases in Ik-1 and MZF1 contribute significantly to the pathogenesis of NPM-ALK^+^ T-cell lymphoma through the upregulation of IGF-IR expression. These findings could be exploited to devise new strategies to eradicate this lymphoma.

**Electronic supplementary material:**

The online version of this article (doi:10.1186/s12943-015-0324-2) contains supplementary material, which is available to authorized users.

## Background

The type I insulin-like growth factor receptor (IGF-IR) tyrosine kinase is a homodimeric protein that is composed of 2 extracellular α and 2 transmembranous β subunits connected by disulfide bonds. IGF-IR belongs to the insulin receptor family whose members exhibit a common structural characteristic in the form of an amino acid motif (Y*XXX*YY) within the activation loop of their respective kinase domains [1,2]. Ligand stimulation of IGF-IR causes its dimerization and phosphorylation, and subsequent activation of downstream signaling systems. Animal models have demonstrated the physiological contributions of IGF-IR to prenatal and postnatal normal cellular homeostasis through interactions with the growth hormone [3,4]. Thus, basal levels of activation of IGF-IR are required for the proliferation and growth of various types of cells, tissues, and organs [4-7]. The critical roles of IGF-IR in early development are illustrated by the *Igf1r-*null mice, which develop generalized organ hypoplasia, delayed bone ossification, and abnormalities in the central nervous system. The *Igf1r*-null mice die prematurely due to lung underdevelopment accompanied by respiratory failure [8].

In addition to its physiological roles, pathological activation of IGF-IR induces cellular transformation and protection from apoptosis-- prerequisites for the establishment and growth of malignant tumors [9-15]. Indeed, IGF-IR is aberrantly overexpressed in and contributes to the survival of a variety of aggressive solid tumors as well as different types of myeloid, lymphoid, and plasma cell neoplasms and, therefore, it may represent an important therapeutic target [16-21]. IGF-IR induces its oncogenic effects through interactions with the downstream survival effectors IRS-1/PI3K/AKT, Grb/Ras/MAPK, and JAK/STAT [22-25].

The mechanisms underlying increased expression of IGF-IR in cancer cells are not completely understood. For instance, only a few transcription factors have been shown to bind the *IGF-IR* gene promoter (15q26.3) and modulate its activity through stimulation or inhibition. These transcription factors include Sp1, WT1, E2F1, STAT1, and EGR-1 [26-34].

Recently, we identified IGF-IR as a major survival molecule that interacts reciprocally with nucleophosmin-anaplastic lymphoma kinase (NPM-ALK) in NPM-ALK-expressing (NPM-ALK^+^) T-cell lymphoma, an aggressive type of cancer that frequently occurs in children and adolescents [35-37]. Compared with its expression in normal human T lymphocytes and reactive lymphoid tissues, the expression of IGF-IR mRNA and protein is remarkably upregulated in NPM-ALK^+^ T-cell lymphoma cell lines and human tumors [36]. Nonetheless, the mechanisms leading to IGF-IR upregulation in this lymphoma remain to be elucidated. We hypothesized that increased IGF-IR expression may be explained by transcriptional aberrancies that exist inherently in this lymphoma. Our data show that the transcription factors Ikaros isoform 1 (Ik-1) and myeloid zinc finger 1 (MZF1) have lower expressions in NPM-ALK^+^ T-cell lymphoma cell lines and human tumors relative to T lymphocytes. We were able to identify sites located within the *IGF-IR* gene promoter that bind Ik-1 and MZF1. Forced expression of Ik-1 and MZF1 significanty decreased the activity of the *IGF-IR* gene promoter and downregulated IGF-IR mRNA and protein levels in these lymphoma cells. In addition, Ik-1- and MZF1-induced downregulation of IGF-IR was assoicated with decreased NPM-ALK^+^ T-cell lymphoma viability, proliferation, migration, and anchorage-independent colony formation.

## Results

### Ik-1 and MZF1 are potential modulators of ***IGF-IR*** gene expression

The TFSearch, MATCH, and Genomatix algorithms identified multiple potential transcription factors, yet we elected to focus on Ik-1 and MZF1 because their 1) matrix similarity thresholds to bind with the *IGF-IR* gene promoter are > 0.9, which has been predicted collectively by the 3 algorithms [the matrix similarity threshold represents the quality of the match between the transcription factor binding sequence and arbitrary parts of the promoter sequence, and is used to minimize false positive results]; 2) contribution to the transcriptional regulation of *IGF-IR* expression has not been previously described; 3) role in the pathogenesis of NPM-ALK^+^ T-cell lymphoma is not known; and 4) contribution to normal and abnormal hematopoiesis has been established [38-42].

### Expressions of Ik-1 and MZF1 are markedly deceased in NPM-ALK^+^ T-cell lymphoma cell lines and human lymphoma tumors

**Figure 1:**
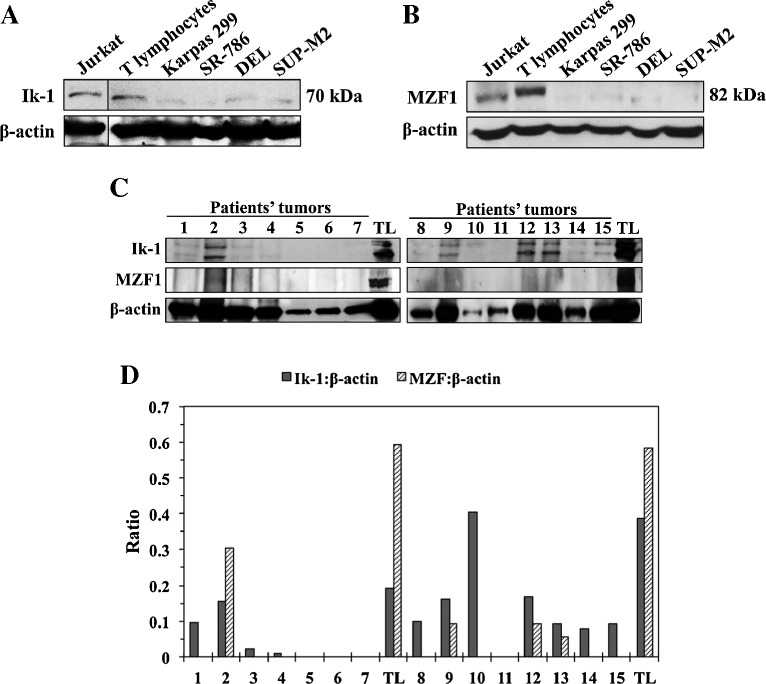
**The expression of Ik-1 and MZF1 is decreased in NPM-ALK** ^**+**^ **T-cell lymphoma and primary tumors from patients. (A)** Western blotting shows that Ik-1 levels were markedly lower in 4 NPM-ALK^+^ T-cell lymphoma cell lines than in T lymphocytes. Jurkat cells were used as a positive control. β-actin showed equal protein loading. Vertical lines have been inserted to indicate repositioned gel lanes. **(B)** Similarly, MZF1 levels were substantially lower in the lymphoma cell lines than in normal human T-cells. Jurkat cells served as a positive control. β-Actin demonstrated equal levels of loaded proteins. **(C)** The expression of Ik-1 and MZF1 is shown in 15 ALK^+^ T-cell lymphoma specimens from patients. Patient’s lysates were divided into 2 groups – 1–7 (left panel) and 8–15 (right panel) – and lysates from 2 different pools of normal human T lymphocytes (TL) were analyzed simultaneously as controls with the corresponding lysates from the patient tumors. Because the quality of the formalin-fixed and paraffin-embedded tissue sections varied significantly, β-actin showed unequal protein levels. **(D)** Despite apparent differences in protein levels loaded in the different lanes, densitometric analysis of the Ik-1/MZF1:β-actin ratio of the Western blot bands shown in (C) supports that the expression of Ik-1 and MZF1 was markedly reduced in 87% and 100%, respectively, of the ALK^+^ T-cell lymphoma specimens from patients compared to human TL. The Western blotting assays were performed 3 independent times with consistent results.

We used Western blotting to screen the expression of Ik-1 and MZF1 proteins in 4 NPM-ALK^+^ T-cell lymphoma cell lines (Karpas 299, SR-786, DEL, and SUP-M2) as well as in normal human T lymphocytes. Jurkat cells were used as a positive control. Ik-1 and MZF1 expressions were remarkably lower in the cell lines than in the human T lymphocytes (Figure 1A and B). To examine the expression of Ik-1 and MZF1 proteins in formalin-fixed and paraffin-embedded ALK^+^ T-cell lymphoma tissues from patients, we initially attempted using immunohistochemical (IHC) staining. However, commercially available Ik-1 antibodies that were suitable for IHC were nonspecific because they detect, not only the Ik-1 protein, but other Ikaros isoforms as well. In addition, we found only one commercially available MZF1 antibody that was listed as suitable for IHC. Our repeated attempts to optimize this antibody for IHC failed because it showed inconsistent results in positive and negative control tissues. Thus, we resorted to using Western blotting to analyze the expression of Ik-1 and MZF1 in protein extracts from 15 ALK^+^ T-cell lymphoma patient tumor sections. Ik-1 and MZF1 were significantly decreased in 87% and 100% of patient samples, respectively (Figure 1C). Densitometric analysis of the Western blotting bands in patient specimens is shown confirming the results depicted in Figure 1C (Figure 1D).

### Interaction and physical association between Ik-1/MZF1 and the ***IGF-IR*** gene promoter

**Figure 2:**
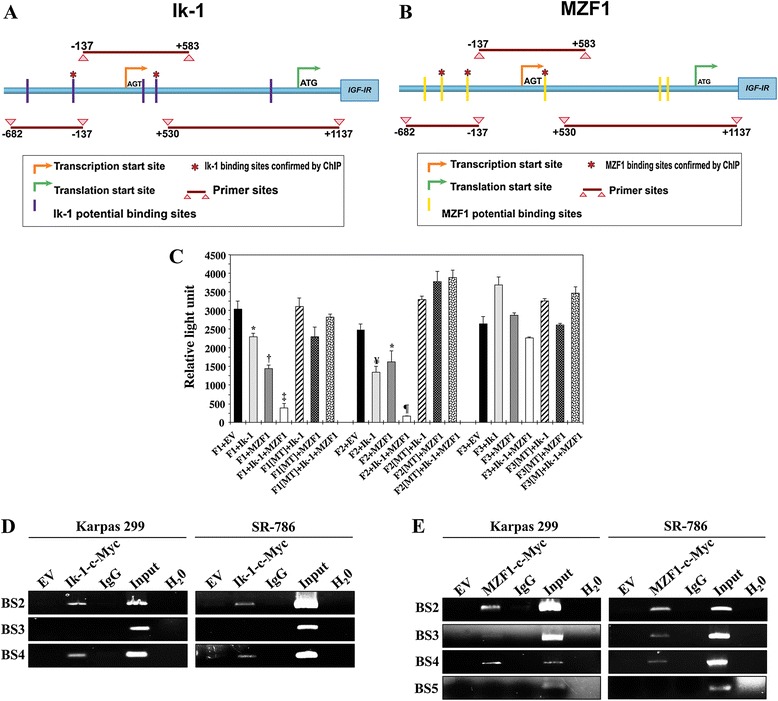
**Ik-1 and MZF1 interact with and bind the** ***IGF-IR*** **gene promoter. (A)** *IGF-IR* gene promoter map including the transcription (AGT) and translation (ATG) start sites. The 3 PCR fragments used in the luciferase assay (F1: −682/-137, F2: −137/+583, F3: +530/+1137) and 5 sites that potentially bind with Ik-1 are shown. *: 2 sites confirmed by ChIP assay. **(B)** In addition to the 3 PCR fragments, 6 sites that potentially bind with MZF1 are depicted. *: 3 sites confirmed by the ChIP. **(C)** Luciferase assay performed in R^−^ cells demonstrates that transfection of Ik-1 or MZF1 decreased *IGF-IR* promoter activity with F1 and F2, but not with F3. Cotransfection of Ik-1 and MZF1 induced more pronounced inhibitory effects on F1 and F2 than one transcription factor alone (*: *P* < 0.05 vs. F1+EV and F2+EV; †: *P* < 0.01 vs. F1+Ik-1 and *P* < 0.0001 vs. F1+EV; ‡: *P* < 0.0001 vs. F1+EV and F1+Ik-1 and *P* < 0.01 vs. F1+MZF1; ¥: *P* < 0.001 vs. F2+EV; ¶: *P* < 0.001 vs. F2+EV and < 0.0001 vs. F2+Ik-1 and F2+MZF1). Ik-1 and MZF1 failed to induce similar effects when F1 and F2 were mutated (MT) at potential binding sites. The results are shown as means ± SE of 3 consistent experiments. **(D)** ChIP assay shows that Ik-1 binds with binding site 2 (BS2) and BS4, but not BS3, of the promoter. Controls, including EV, IgG, input, and H_2_O, worked properly. The 2 binding sites are identified by an (*) in (A). **(E)** ChIP assay confirms that MZF1 binds with the promoter at BS2, BS3, and BS4, and not BS5, which are marked by an (*) in (B). Controls worked properly. Some of the panels shown in (D) and (E) have been slightly enhanced in their entirety to assist with the visualization of the weak bands, which are pertinent to the results.

To elucidate whether Ik-1 and MZF1 can bind to and regulate the *IGF-IR* gene promoter, we performed a luciferase assay in R^−^ cells, mouse 3 T3-like fibroblasts with targeted ablation of *Igf1r* gene [8], using 3 different fragments of the human *IGF-IR* promoter construct (F1: −682/-137, F2: −137/+583, F3; +530/+1137; illustrated in Figure 2A and B) along with either EV, Ik-1 or MZF1 expression plasmids. We also performed the luciferase assay using mutated versions of the promoter fragments that were sequentially deleted at the corresponding binding sites. Ik-1 and MZF1 significantly decreased the luciferase activity of the F1 and F2, but not the F3, regions of the human *IGF-IR* promoter (Figure 2C). The inhibitory effects on F1 and F2 were much more pronounced when R^−^ cells were simultaneously transfected with Ik-1 and MZF1 (Figure 2C). These effects did not occur when mutated F1 and F2 were used (Figure 2C). Our results suggest that Ik-1 and MZF1 can downregulate *IGF-IR* gene promoter through interactions with binding sites located within the F1 and F2 regions.

Using the 3 web-based transcription factor search algorithms, we were able to predict 5 sites within the human *IGF-IR* promoter that could potentially bind with Ik-1 and 6 sites that could potentially bind with MZF1 (Figure 2A and B). To determine which of these sites is responsible for Ik-1 or MZF1 interactions with the *IGF-IR* promoter, we performed a ChIP assay. Because there are very low levels of endogenous Ik-1 and MZF1 in Karpas 299 and SR-786 cells, c-Myc-Ik-1 and c-Myc-MZF1 expression constructs were used. Using c-Myc antibody, the transcription factors were immunoprecipitated and the sonicated chromatin was used to perform an RT-PCR analysis using primers flanking the potential binding sites for Ik-1 and MZF1 within the human *IGF-IR* promoter. The ChIP assay identified 2 binding sites (BS2 and BS4) for Ik-1 and 3 binding sites for MZF1 (BS2, BS3, and BS4) (Figure 2D, 2E; these sites are depicted in Figure 2A and B). The RT-PCR (2 different sets of primers were tried) showed nonspecific binding in the ChIP assay for Ik-1 BS1 and BS5, and for MZF1 BS1 and BS6 (data not shown). The nonspecific binding could be attributed to the fact that these regions contained highly repetitive sequences.

### Ectopic expression of Ik-1 and MZF1 in NPM-ALK^+^ T-cell lymphoma cells downregulates IGF-IR mRNA and protein levels and decreases the phosphorylation of downstream targets

**Figure 3:**
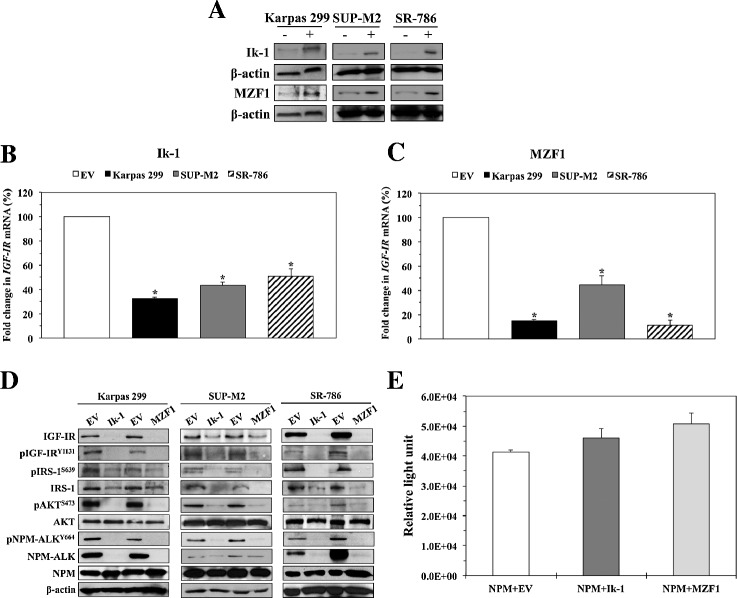
**Ik-1 and MZF1 decrease IGF-IR mRNA and protein levels and the phosphorylation of downstream targets. (A)** Western blotting demonstrates increased expression levels of Ik-1 and MZF1 proteins at 48 h after transfection into 3 NPM-ALK^+^ T-cell lymphoma cell lines. β-actin shows equal protein loading (−: transfection of EV; +: transfection of Ik-1 or MZF1). **(B)** Transfection of Ik-1 remarkably decreased *IGF-IR* mRNA levels in Karpas 299, SUP-M2, and SR-786 cell lines (*: < 0.0001 compared with EV). **(C)** Similarly, transfection of MZF1 induced a significant decrease in *IGF-IR* mRNA levels (*: < 0.0001 compared with EV). The results depicted in (B) and (C) represent the means ± SE of 3 experiments. **(D)** Western blotting shows that transfection of Ik-1 and MZF1 into Karpas 299, SUP-M2, and SR-786 cell lines induced marked downregulation of IGF-IR protein, which was associated with decreased pIGF-IR levels. Moreover, the decrease in IGF-IR/pIGF-IR expression levels was associated with decreased phosphorylation of important IGF-IR targets including IRS-1, AKT, and NPM-ALK. Whereas basal levels of AKT remained unchanged, notable decrease in IRS-1 protein was observed. The 3 web-based transcription factor search algorithms showed that Ik-1 and MZF1 could potentially bind the *IRS-1* gene promoter. In contrast, searching these algorithms did not support potential binding of Ik-1 or MZF1 and the *NPM* gene promoter, where the expression of NPM-ALK protein is regulated at the transcriptional level. **(E)** In line with lack of potential binding/interaction between Ik-1 or MZF1 and the *NPM* gene promoter, a luciferase assay performed in R^−^ cells shows that transfection of Ik-1 and MZF1 does not decrease the *NPM* promoter activity. The results represent means ± SE of 3 consistent experiments.

We next determined the effect of ectopic expression of Ik-1 and MZF1 on IGF-IR mRNA and protein levels. Transfection of Karpas 299, SUP-M2, and SR-786 cells with Ik-1 and MZF1 expression plasmids, but not EV, increased adequately the level of expression of the corresponding transcription factor (Figure 3A). In further support of their regulatory effects on *IGF-IR* gene expression, Ik-1 and MZF1 significantly decreased *IGF-IR* mRNA levels (Figures 3B, C). The decrease in *IGF-IR* mRNA was associated with downregulation of IGF-IR protein expression, as well as levels of its activated/phosphorylated form, pIGF-IR (Figure 3D). Ik-1- and MZF1-induced downregulation of IGF-IR and pIGF-IR was associated with a significant decrease in the phosphorylation of important downstream targets such as pAKT and pIRS-1 (Figure 3D). Whereas there was no notable decrease in total AKT levels, Ik-1 and MZF1 decreased total IRS-1 protein levels (Figure 3D). When the *IRS-1* gene promoter sequence was analyzed using the 3 web-based transcription factor search algorithms, we discovered that Ik-1 and MZF1 have the potential to bind to the *IRS-1* gene promoter, which may explain this unexpected finding.

### Ectopic expression of Ik-1 and MZF1 in NPM-ALK^+^ T-cell lymphoma cells is associated with downregulation of NPM-ALK protein levels

We have recently demonstrated that IGF-IR binds and interacts with NPM-ALK, and through their binding/interactions, IGF-IR appears to maintain the stability of the NPM-ALK protein [36,37]. Consistent with our earlier observations, Ik-1- and MZF1-induced downregulation of IGF-IR was associated with a marked decrease in the expression of NPM-ALK protein (Figure 3D). To further explore this finding, the 3 web-based transcription factor search algorithms were utilized and each failed to predict potential binding sites for Ik-1/MZF1 with the *NPM* gene promoter, where the promoter region for the transcriptional regulation of the *NPM-ALK* chimeric oncogene resides [43]. In line with this observation, Western blot analysis showed no decrease in the levels of wild-type NPM protein after transfection of Ik-1 and MZF1 in NPM-ALK^+^ T-cell lymphoma cell lines (Figure 3D). In addition, a luciferase assay in R^−^ cells failed to demonstrate decreased *NPM* gene promoter activity after transfection of Ik-1 or MZF1 (Figure 3E). These results indicate that, most likely, an indirect mechanism of NPM-ALK downregulation exists through Ik-1 and MZF1-mediated suppression of IGF-IR expression.

### Ik-1 and MZF1 decrease the viability, proliferation, migration, and anchorage-independent colony formation of NPM-ALK^+^ T-cell lymphoma cells

**Figure 4:**
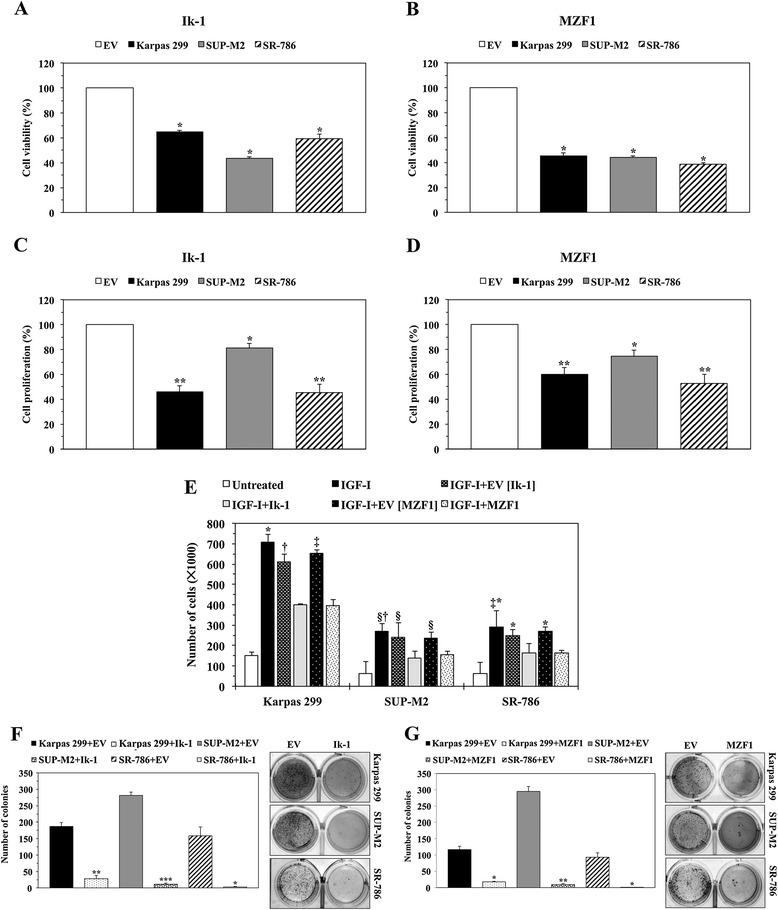
**Transfection of Ik-1 and MZF1 decreases the viability, proliferation, migration, and anchorage-independent colony formation of NPM-ALK** ^**+**^ **T-cell lymphoma cells. (A)** Compared with EV, transfection of Ik-1 decreased the viability of the lymphoma cells at 48 h (*: *P* < 0.0001). **(B)** In a similar fashion, transfection of MZF1 decreased cellular viability (*: *P* < 0.0001). **(C)** Ik-1 decreased cellular proliferation (*: *P* < 0.05; **: *P* < 0.01). **(D)** Transfection of MZF1 also reduced the proliferation of these cells (*: *P* < 0.05; **: *P* < 0.01). **(E)** IGF-I stimulated the migration of the lymphoma cells. Whereas this effect was substantially decreased when cells were treated with IGF-I and transfected with Ik-1 or MZF1; EV failed to induce similar effects (Karpas 299, *: *P* < 0.0001 in IGF-I vs. IGF-I+Ik-1 and IGF-I+MZF1, †: *P* < 0.01 in IGF-I+EV [Ik-1] vs. IGF-I+Ik-1, ‡: *P* < 0.001 in IGF-I+EV [MZF1] vs. IGF-I+MZF1; SUP-M2, §: *P* < 0.05 in IGF-I vs. IGF-I+Ik-1, IGF-I+EV [Ik-1] vs. IGF-I+Ik-1 and IGF-I+EV [MZF1] vs. IGF-I+MZF1, †: *P* < 0.01 in IGF-I vs. IGF-I+MZF1; SR-786, *: *P* < 0.0001 in IGF-I vs. IGF-I+MZF1, IGF-I+EV [Ik-1] vs. IGF-I+Ik-1, and IGF-I+EV [MZF1] vs. IGF-I+MZF1, ‡: *P* < 0.001 in IGF-I vs. IGF-I+Ik-1). **(F)** Ik-1 abrogated anchorage-independent colony formation of the lymphoma cells at 7 days after transfection (*: *P* < 0.05; **: *P* < 0.01; ***: *P* < 0.001 compared with EV). The numbers of colonies are shown in the left panel, and examples of the colonies are illustrated in the right panel. **(G)** Similarly, MZF1 halted the lymphoma cell colony formation (*: *P* < 0.05; **: *P* < 0.01 compared with EV). The numbers of colonies are shown in the left panel, and examples of the colonies are depicted in the right panel. Results represent means ± SE of at least 3 consistent experiments.

We performed a series of cellular assays to test the biological impact of forced expression of Ik-1 and MZF1 in NPM-ALK^+^ T-cell lymphoma. Transfection of Ik-1 or MZF1, but not EV, significantly decreased the viability, proliferation, and migration of Karpas 299, SR-786, and SUP-M2 cells (Figure 4A,B,C,D and E). Ik-1 and MZF1 also abrogated anchorage-independent colony formation of NPM-ALK^+^ T-cell lymphoma cells (Figure 4F,G).

### ***IGF-IR*** mRNA demonstrates prolonged decay time in NPM-ALK^+^ T-cell lymphoma

**Figure 5:**
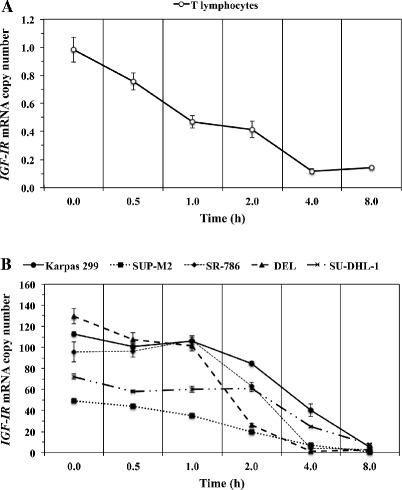
***IGF-IR*** **mRNA expressed in NPM-ALK** ^**+**^ **T-cell lymphoma cells exhibits prolonged decay time compared with** ***IGF-IR*** **mRNA from normal human T lymphocytes. (A)** The decay of *IGF-IR* mRNA in normal human T lymphocytes over 8 h is illustrated. The 50% level (t_1/2_) of *IGF-IR* mRNA was detected at 0.8 h. **(B)** In contrast with T lymphocytes, the NPM-ALK^+^ T-cell lymphoma cell lines expressed remarkably higher basal levels of *IGF-IR* mRNA, with DEL and SUP-M2 cells demonstrating the highest and lowest levels, respectively. The t_1/2_ for *IGF-IR* mRNA level was achieved after longer periods of time in the lymphoma cells than in normal T lymphocytes (SU-DHL-1: 3.7 h, Karpas 299: 3.4 h, SR-786: 2.6 h, SUP-M2: 1.9 h, DEL: 1.5 h). The mean of the t_1/2_ *IGF-IR* mRNA decay time in the lymphoma cells was 2.62 ± 0.4 h (SE). Results shown represent the means ± SE of 3 experiments.

Because the mechanisms leading to aberrant expression of oncogenes and tumor suppressor genes are typically executed at more than one regulatory level, we questioned whether aberrancies in *IGF-IR* mRNA decay also exist in this lymphoma. Consistent with our previously published results [36], basal levels of *IGF-IR* were much higher in the NPM-ALK^+^ T-cell lymphoma cells than in the normal human T lymphocytes (Figures 5A, B). In T lymphocytes, *IGF-IR* mRNA decreased to 50% of its basal levels (t_1/2_) at 0.8 h (Figure 5A). The decay of *IGF-IR* mRNA varied among 5 different NPM-ALK^+^ T-cell lymphoma cell lines, yet it consistently occurred at a more prolonged rate than T lymphocytes (Figure 5B; t_1/2_ mean ± SE: 2.62 ± 0.4 h).

### NPM-ALK does not influence the expression levels of IGF-IR or IGF-I

**Figure 6:**
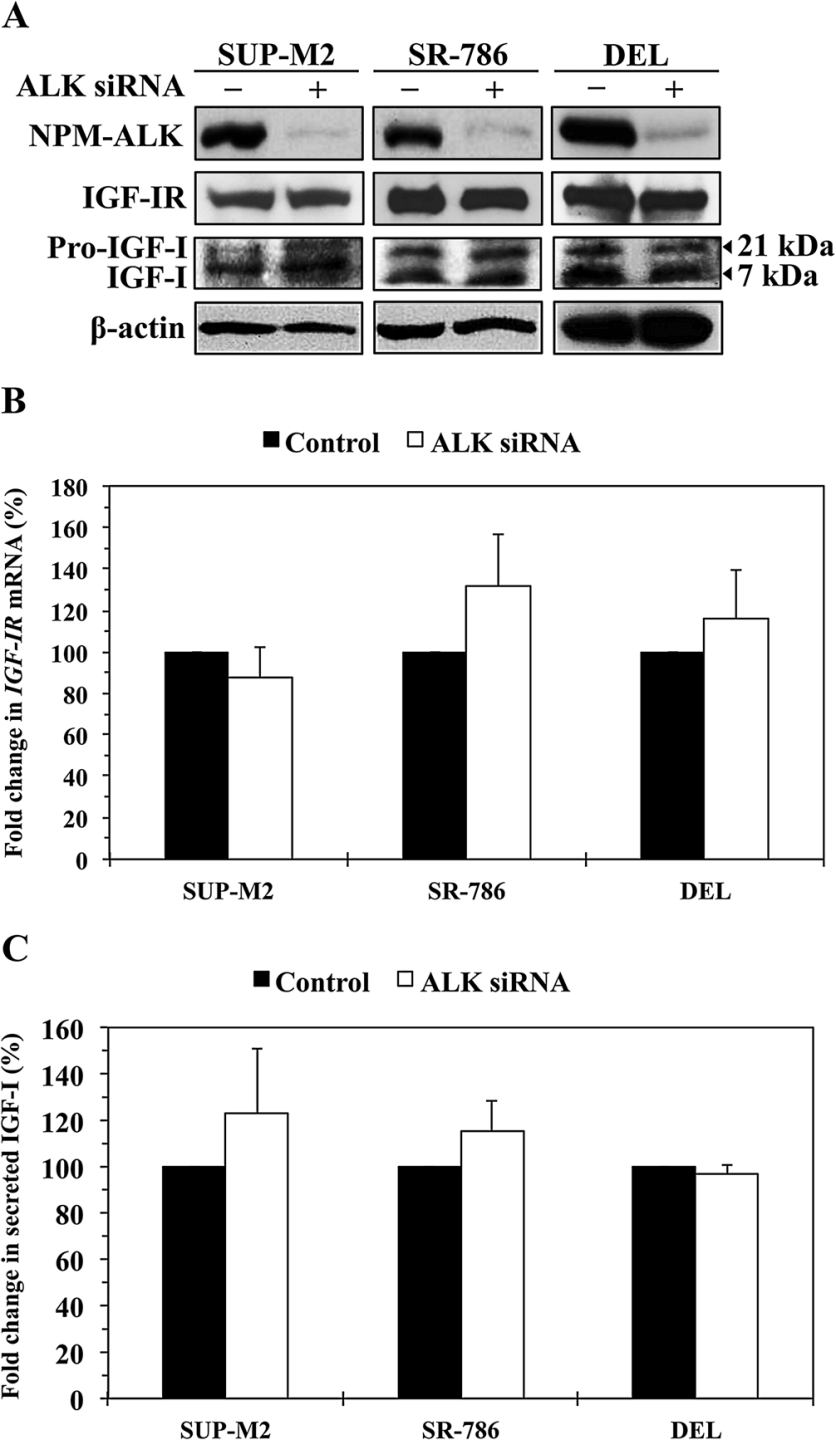
**NPM-ALK oncogenic protein does not affect the levels of expression of IGF-IR and IGF-I. (A)** Western blotting shows that at 48 h, downregulation of NPM-ALK by ALK siRNA was not associated with decreased expression of IGF-IR, pro-IGF-I or IGF-I proteins in SUP-M2, SR-786, and DEL cell lines. β-Actin shows equal protein loading. Analysis of IGF-IR levels after transfection of the cells with ALK siRNA was performed at extended time points (12, 24, 48, 72, and 96 h), and also in other cell lines including Karpas 299 and SU-DHL-1, with similar results (data not shown). **(B)** Downregulation of NPM-ALK in the 3 cell lines did not decrease the levels of *IGF-IR* mRNA. The example shown is at 48 h after transfection of the cells with ALK siRNA. The results are shown as means ± SE of 4 consistent experiments. In addition, analysis of *IGF-IR* mRNA was performed at other time points and cell lines as described in (A). Changes in *IGF-IR* mRNA levels were not detected at any time point (data not shown). **(C)** An ELISA assay showing that specific downregulation of NPM-ALK did not significantly decrease the levels of IGF-I secreted from the NPM-ALK^+^ T-cell lymphoma cells. The results represent the means ± SE of 3 experiments.

To explore whether NPM-ALK plays a role in increased levels of expression of IGF-IR, we transfected SUP-M2, SR-786, and DEL cells with ALK siRNA for extended time periods. Although ALK siRNA effectively downregulated NPM-ALK (Figure 6A), levels of IGF-IR protein (Figure 6A) and mRNA (Figure 6B) remained unchanged. In addition, decreased NPM-ALK was not associated with decreased endogenous pro-IGF-I or IGF-I (Figure 6A) or decreased secreted IGF-I (Figure 6C).

### ***IGF-IR*** gene is not amplified in NPM-ALK^+^ T-cell lymphoma

FISH assay failed to support amplification of *IGF-IR* gene in NPM-ALK^+^ T-cell lymphoma cells (Additional file 1: Figure S1-AF and Table S1-AF).

## Discussion

In this paper we show that previously unidentified defects in transcriptional machinery contribute to the pathogenesis of NPM-ALK^+^ T-cell lymphoma. Compared with their expression in normal human T lymphocytes, the transcription factors Ik-1 and MZF1 are markedly decreased in NPM-ALK^+^ T-cell lymphoma cell lines and lymphoma tumors from patients. Substantial evidence is provided to support that Ik-1 and MZF1 possess ability to bind specific sites residing within the *IGF-IR* gene promoter and inhibit its activity. In agreement with these observations, ectopic expression of Ik-1 and MZF1 in NPM-ALK^+^ T-cell lymphoma cells causes remarkable downregulation of the expression of IGF-IR mRNA and protein. Also, transfection of Ik-1 and MZF1 was associated with decreased cell viability, proliferation, migration, and anchorage-independent colony formation of NPM-ALK^+^ T-cell lymphoma cells, asserting the tumor-suppressing impact of Ik-1 and MZF1 in this lymphoma. It has been previously shown that Ik-1 induces tumor suppressor effects primarily in hematopoietic cellular elements [44-46]; nonetheless, the contribution of MZF1 to tumorigenesis is more diverse as it may induce oncogenic or tumor suppressor effects in hematopoietic and non-hematopoietic cells [47-52].

The *IGF-IR* gene promoter is tightly regulated during mammalian development, and during the embryonic and early postnatal stages it induces the transcription of high levels of *IGF-IR* mRNA, which decrease to much lower levels during growth [53]. The *IGF-IR* gene promoter is a TATA-less and CAAT-less promoter, and like other structurally similar promoters, the *IGF-IR* promoter is GC-rich [26,27,54]. The transcription of the *IGF-IR* gene is therefore initiated from a unique site contained within an “initiator” motif similar to the ones present in the terminal deoxynucleotidyl transferase and adenovirus middle late gene promoters [27,28,55].

Although levels of expression of IGF-IR during physiological and pathological conditions can be rigorously determined at the transcriptional level [56], thus far relatively few transcription factors have been shown to be capable of binding with and regulating the expression of the *IGF-IR* gene promoter. The multiple GC boxes present in this promoter form potential binding sites for members of the zinc-finger transcription factor family. In line with this notion, earlier studies using rat *IGF-IR* gene promoter showed that Sp1, a zinc-finger transcription factor, binds with GC boxes located within the 5′-flanking region and one homopurine/homopyrimidine motif (CT element) in the 5′-untranslated region of *IGF-IR* gene promoter to enhance its activity [28,29]. The *IGF-IR* gene promoter also includes *cis*-elements for members of the early growth response family of zinc-finger proteins including the WT1 Wilms’ tumor suppressor, which, in contrast with Sp1, downregulates the expression of *IGF-IR* [30,31]. Indeed, increased expression of WT1 protein was associated with a reciprocal decrease in the expression of IGF-IR protein and receptor number in prostate cancer cells, and downregulation of WT1 increased IGF-IR expression in glioblastoma [57,58]. Albeit less extensively studied, E2F1 and EGR-1 are also implicated in the positive regulation, and STAT1 in the negative regulation of *IGF-IR* gene expression [32-34].

In addition to the direct regulatory effects of Sp1 and WT1, several studies have elucidated indirect contributions of oncogenic and tumor suppressor proteins to the regulation of the *IGF-IR* gene expression through interactions with these 2 transcription factors. In breast cancer, BRCA1 appears to suppress the *IGF-IR* promoter activity, but there is no evidence to support BRCA1’s binding and direct interactions with the *IGF-IR* promoter. Instead, BRCA1 most likely suppresses the activity of the *IGF-IR* promoter through the sequestration of Sp1 [59,60]. Similarly, in breast cancer cells, the estrogen receptor enhances *IGF-IR* gene promoter activity via interactions with Sp1 and WT1 [61,62]. Also, MCF7 breast cancer cells that express caveolin-1 demonstrate much higher levels of *IGF-IR* gene promoter activity, and the effects of caveolin-1 on the *IGF-IR* gene promoter were mediated through Sp1 [63]. Furthermore, the tumor suppressor transcription factor Kruppel-like factor 6 (KLF6) activates *IGF-IR* gene transcription through synergy with Sp1 [64]. Moreover, it was found that wild-type p53 downregulates *IGF-IR* gene expression and mutated p53 enhances this expression [65-67]. The regulatory mechanisms conferred by p53 also do not involve specific binding with the *IGF-IR* gene promoter but seem to be mediated, at least partially, by protein-protein interactions between p53 and Sp1.

The *NPM-ALK* chimeric oncogene plays a central role in the survival of NPM-ALK^+^ T-cell lymphoma [35]. We have previously demonstrated that NPM-ALK and IGF-IR reciprocally collaborate to sustain their high phosphorylation levels in this lymphoma [36,37]. Here we questioned whether NPM-ALK, similar to oncogenic proteins described above, possesses regulatory capacity pertinent to IGF-IR expression. Our results show that specific abrogation of NPM-ALK by siRNA failed to reduce IGF-IR protein or mRNA levels. Furthermore, it was previously demonstrated that chimeric oncogenes such as the Ewing sarcoma fusion proteins induce the expression of IGF-I, the primary ligand of IGF-IR [68]. Our data indicate that endogenous and secreted IGF-I levels are most likely regulated independently from NPM-ALK. Collectively, these results suggest that the effects of NPM-ALK on IGF-I/IGF-IR signaling are mediated post-translationally through phosphorylation of IGF-IR protein [36,37].

We have also previously demonstrated that NPM-ALK and IGF-IR are physically associated, and it appears that this physical association, through interactions with Hsp90, enhances the stability of NPM-ALK protein [37]. In the current study, the decrease in IGF-IR expression after Ik-1 and MZF1 transfection was also associated with pronounced decrease in NPM-ALK basal protein levels. Although these results agree with our previous observations, we sought to investigate whether Ik-1 or MZF1 is capable of regulating the expression of NPM-ALK directly at the transcriptional level. The web-based transcription factor search algorithms failed to predict potential binding sites between the *NPM* gene promoter, where the transcription of the *NPM-ALK* chimeric oncogene is driven [43], and either Ik-1 or MZF1. Furthermore, a luciferase assay using an *NPM* reporter construct showed that transfection of Ik-1 and MZF1 does not affect *NPM* promoter activity or protein levels. Therefore, our current results indicate that the decrease in NPM-ALK protein levels occurs secondarily to Ik-1- and MZF-1-induced downregulation of IGF-IR protein expression.

The Ik-1 and MZF1 transcription factors play physiological roles in the development of normal hematopoiesis [39-42]. In the present paper we describe for the first time in any type of cancer cells the negative regulation of *IGF-IR* gene expression by Ik-1 and MZF1 transcription factors. Ik-1 regulates transcription by binding to specific consensus binding sites (C/TGGGAA/T) within target promoters [69]. Similarly, MZF1’s 13 zinc fingers are separated into 2 arms, and each arm has the ability to independently bind to specific binding sites within the promoters of target genes: the first domain of fingers 1–4 (ZN 1–4) binds to the sequence 5′-AGTGGGGA-3′, and the second domain of fingers 5–13 (ZN 5–13) binds the core sequence 5′-CGGGnGAGGGGGAA-3′ [41]. Similar to other transcription factors that bind with *IGF-IR* gene promoter, we found that Ik-1 and MZF1 possess the potential to bind with sequences located both upstream and downstream of the transcription start site within the 5′-flanking region as well as within the 5′-untranslated region. Specifically, potential binding sites for Ik-1 are located at nucleotides −504/-491, −*138/-125*, +77/+90, *+427/+440*, and +1011/+1024, and potential binding sites for MZF1 are located at nucleotides −504/-496, *−299/-291*, *−138/-130*, *+501/+514*, +919/+928, and +1011/+1019 (binding sites confirmed by ChIP are in italics). To our knowledge, these binding sites have not been previously described to bind with any of the transcription factors that are known to regulate *IGF-IR* gene. Among the previously described transcription factors, Sp1, E2F1, and EGR-1 showed a greater net change in promoter activity at binding sites located downstream of the transcription start site [29,33,34]. While this pattern was similar to Ik-1, MZF1 demonstrated a greater net change in *IGF-IR* promoter activity at binding sites located upstream of the transcription start site, which resembles the inhibitory effects induced by WT1 [30].

Ik-1- and MZF1-induced downregulation of IGF-IR was associated with decreased levels of its activated/phosphorylated form, pIGF-IR. These effects induced downregulation of the phosphorylation levels of the molecular targets of IGF-IR including IRS-1, AKT, and NPM-ALK. Whereas basal levels of AKT remained unchanged, the basal levels of IRS-1 decreased after transfection of Ik-1 and MZF1. To further analyze this unexpected finding, we searched the web-based transcription factor algorithms and found that the *IRS-1* gene promoter contains sites that could potentially function as targets for Ik-1 and MZF1 transcriptional activity. It is important to mention that IRS-1 is also a downstream target of NPM-ALK phosphorylation activity [70]. Although further analysis is required to support this idea, we cannot completely rule out that Ik-1 and MZF1 act as tumor suppressors in this lymphoma through targeting the expression of IGF-IR and IRS-1.

It is important, however, to emphasize that deregulated systems underlying the pathogenesis of NPM-ALK^+^ T-cell lymphoma are complex owing to the fact that they originate from more than one defected regulatory mechanism [35]. Although our results provide strong evidence that the aberrant decrease in the expression of Ik-1 and MZF1 transcription factors contributes to upregulation of an important oncogenic protein, i.e., IGF-IR, we elected to investigate whether other transcriptional or posttranscriptional mechanisms exist to further enhance IGF-IR expression. Our experiments failed to support the presence of *IGF-IR* gene amplification, an aberrant transcriptional mechanism, in NPM-ALK^+^ T-cell lymphoma. *IGF-IR* gene amplification has been previously reported in small subgroups of patients with solid tumors such as lung cancer and gastrointestinal stromal tumors [71,72]. Nonetheless, we also found that the posttranscriptional decay of *IGF-IR* mRNA in NPM-ALK^+^ T-cell lymphoma occurs over a remarkably prolonged time compared with the decay of *IGF-IR* mRNA that is physiologically expressed in human T lymphocytes. To this end, our data suggest a model in which upregulation of IGF-IR in NPM-ALK^+^ T-cell lymphoma results from multilevel defects in transcriptional and posttranscriptional mechanisms, which reflects the complexity of survival signaling in this lymphoma.

## Conclusions

The vast majority of the previously published literature has implicated overexpression of several transcription factors in the tumor-promoting effects in NPM-ALK^+^ T-cell lymphoma [35,73-78]. In this report, we were able to show that substantial decreases in the Ik-1 and MZF1 transcription factors play important roles in the pathogenesis of this lymphoma. In this capacity, Ik-1 and MZF1 are physiologically capable of binding with the *IGF-IR* gene promoter to inhibit its transcriptional activity, which induces downregulation of the expression of IGF-IR oncogenic tyrosine kinase. Collectively, lack of these molecular events supports the survival of NPM-ALK^+^ T-cell lymphoma. Notably, the upregulation of IGF-IR expression appears to be independent from NPM-ALK expression. However, we cannot completely exclude that the outcome of decreased expression of Ik-1 and MZF1 in this lymphoma is not only mediated through upregulation of IGF-IR because it is possible that these 2 transcription factors are involved in the regulation of other survival/oncogenic proteins. The findings presented herein are expected to further advance current understanding of the pathobiology of NPM-ALK^+^ T-cell lymphoma and may contribute to the development of novel therapeutic approaches to efficiently eradicate this aggressive cancer.

## Materials and methods

### Web-based transcription factor search algorithms

To identify transcription factors that can potentially bind to the human *IGF-IR* gene promoter, 3 web-based transcription factor search algorithms were used: Genomatix (www.genomatix.de), MATCH (www.gene-regulation.com/pub/programs.html), and TFSearch (we used this transcription factor search algorithm when we initiated the study, but we noticed that now this algorithm has been removed and the website is not available for online support. Importantly, the findings we obtained from TFSearch matched exactly the findings gathered from Genomatix and MATCH).

### Cell lines

Five NPM-ALK^+^ T-cell lymphoma cell lines were used in this study: Karpas 299, SUP-M2, SR-786, DEL, and SU-DHL-1 (DSMZ, Braunschweig, Germany). The R^−^ cell line (mouse 3 T3-like fibroblasts with targeted ablation of *Igf1r* gene [8]; gift from Dr. Renato Baserga, Thomas Jefferson University, Philadelphia, PA) was used as the host cell line for luciferase assay studies. Normal human peripheral blood CD3^+^ pan-T lymphocytes were used in some experiments (catalog number: PB0091F; StemCell Technologies, Vancouver, BC, Canada). In addition, Jurkat cells (ATCC, Manassas, VA) were used as a positive control for the expression of Ik-1 and MZF1 [38,79]. The T lymphocytes and the Jurkat and NPM-ALK^+^ T-cell lymphoma cell lines were maintained in RPMI 1640 medium supplemented with 10% FBS (Sigma, St. Louis, MO), glutamine (2 mM), penicillin (100 U/mL), and streptomycin (100 μg/mL) at 37°C in humidified air with 5% CO_2_. DMEM supplemented with 10% FBS was used to culture the R^−^ cells under the same conditions.

### Antibodies

The following antibodies were used: pIGF-IR^Y1131^ (3021), pALK^Y1604^ (Y664 in NPM-ALK; 3341), pAKT^S473^ (4051), and Ikaros (5443) (Cell Signaling Technology, Danvers, MA); IRS-1 (ab40777), AKT (ab8805), NPM (ab52644), and MZF1 (ab64866) (Abcam, Cambridge, MA); IGF-IR (396700; Life Technologies, Grand Island, NY); pIRS-1^S639^ (sc-22300; Santa Cruz Biotechnology, Santa Cruz, CA); ALK (M719501-2; Dako, Carpinteria, CA); c-Myc (631206, Clontech Laboratories, Mountain View, CA); IGF-I (05–172; Millipore, Billerica, MA); and β-actin (A-5316; Sigma).

### RNA extraction, cDNA synthesis, and relative quantitative PCR (qPCR)

Total RNA was isolated and purified using the RNAeasy Mini Kit (Qiagen). Briefly, 1 × 10^6^ cells were collected by centrifugation at 200 *g* for 5 min, washed twice in phosphate-buffered saline (PBS), and subjected to lysis and homogenization with Buffer RLT using QiaShredder spin columns (Qiagen). Homogenized cells were re-suspended in an equal volume of 70% ethanol and passed through the spin columns. Cells were then washed once using Buffer RW1 and twice using Buffer RPE (Qiagen). Total RNA was collected upon elution with RNase-free water. Optical density was detected using spectrophotometry (NanoDrop 2000, Thermo Fisher Scientific, Waltham, MA).

cDNA synthesis was performed using the Superscript III RT protocol (Invitrogen, Carlsbad, CA). Approximately 0.3 μg total RNA was used for reverse transcription. Briefly, total RNA, oligo deoxy-thymine (dT), and deoxytrinucleotide triphosphate (dNTP) were admixed, and the final volume was adjusted to 10 μL using RNase-free water. The RNA mixture and primer were denatured at 65°C for 5 min and then placed on ice. The master reaction mixture consisting of 10× cDNA synthesis buffer, 0.1 M DTT, RNaseOUT, SuperscriptIII RT, MgCl_2_, and RNase-free H_2_O was prepared on ice and vortexed gently. Then, 10 μL of the reaction mixture was pipetted into each reaction tube on ice. Samples were transferred to a thermal cycler preheated to the appropriate cDNA synthesis temperature and incubated at 50°C for 60 min and then at 85°C for 5 min. Finally, 1.0 μL RNase H was added and the samples were incubated at 37°C for 20 min to remove template RNA.

Relative qPCR was used to measure the levels of *IGF-IR* mRNA in NPM-ALK^+^ T-cell lymphoma cell lines after transfection with Ik-1 or MZF1 expression vectors (Open Biosystems, Pittsburgh, PA) using reactions containing reverse-transcribed cDNA, *IGF-IR* primer/probe, and Taqman Mastermix (Applied Biosystems, Grand Island, NY). 18S ribosomal RNA was used as the endogenous control.

### Transfection

Cells were transfected with Ik-1 or MZF1 expression plasmids using electroporation and the Amaxa 4D Nucleofector System (solution SF, program CA-150; Lonza, Walkersville, MD) and then incubated for 48 h. For luciferase assays, R^−^ cells were transfected using Lipofectamine 2000 reagent. Briefly, 1 × 10^6^ R^−^ cells were seeded in 6-well plates. The following day, plasmids were incubated in 100 μL OptiMEM media for 5 min at room temperature. Simultaneously, 7 μL lipofectamine was incubated in a separate tube. Then, the contents of the plasmid tubes were added to the lipofectamine and incubated for 20 min at room temperature. Finally, the plasmid mixtures were added to the corresponding plate wells containing the R^−^ cells. In some experiments, scrambled or ALK siRNA (Dharmacon, Pittsburgh, PA) was transfected into NPM-ALK^+^ T-cell lymphoma cell lines by using the same approach.

### Construction of the human ***IGF-IR*** gene promoter

Three different fragments of the human *IGF-IR* gene promoter were amplified using genomic DNA (Promega, Madison, WI). Briefly, 500 ng of genomic DNA was added to HotStarTaq plus Q solution additive mixture (Qiagen) and subjected to Touchdown PCR. Primers and amplification conditions are shown in Table 1 and Table 2.

**Table 1:** **Sequence of the primers used to construct the 3 fragments (F1, F2, and F3) of the human** ***IGF-IR*** **gene promoter**

**Primer name**	**Sequence**
**F1** ** (Forward)**	5′-CTC TCC TCG AGC CAC TCT GGG C-3′
**F1** ** (Reverse)**	5′-CAA GAC GTG CGG AGC GGA GC-3′
**F2** ** (Forward)**	5′-TCC GCA CGT CTT GGG GAA CC-3′
**F2** ** (Reverse)**	5′-GCC CCG AAG TCC GGG TCA CA-3′
**F3** ** (Forward)**	5′-GAC TCC GCG TTT CTG CCC CTC-3′
**F3** **(Reverse)**	5′-CTC CAC TCG TCG GCC AGA GC-3′

**Table 2:** **The amplification conditions used in Touchdown PCR for the construction of 3 fragments of the human** ***IGF-IR*** **gene promoter (∞: hold)**

**Phase 1**	**Step**	**Temperature**	**Time**
1	Denature	95°C	15 min
2	Denature	95°C	30 sec
3	Anneal	70°C	45 sec
		−1.0°C^*^	
4	Elongate	72°C	1 min
Repeat steps 2–4 (15 times)			
**Phase 2**	**Step**	**Temperature**	**Time**
5	Denature	95°C	30 sec
6	Anneal	60°C	45 sec
7	Elongate	72°C	1 min
Repeat steps 5–7 (25 times)			
**Termination**	**Step**	**Temperature**	**Time**
8	Elongate	72°C	5 min
9	Halt reaction	4°C	15 min
10	Hold	4°C	∞

^*^Every time steps 2–4 are repeated, the annealing temperature is decreased by 1.0°C/cycle until the estimated melting temperature is reached.

PCR products were run on 1.5% agarose gel, excised, and purified using the Qiaquick Gel Extraction Kit (Qiagen). PCR products were subcloned at a 1:5 molar ratio into the pGEM vector using the TA cloning system (Promega). The ligated products were transformed using MaxEfficiency DH5α-competent cells (Invitrogen) overnight at 37°C, and positive clones were selected and verified by PCR and direct DNA sequencing. Clones containing the correct insert were amplified in ampicillin containing Luria-Bertani broth (Corning Costar, Corning, NY). Plasmids were isolated and purified using the Purelink Quick Plasmid Miniprep Kit (Invitrogen). To construct reporter plasmids containing the human *IGF-IR* gene promoter fragments, the pGEM plasmids and the PGL4.17 luciferase vector (Promega) were subjected to restriction enzyme digestion using ZraI/SpeI (Promega) and EcoICRI/NheI (New England Biolabs, Ipswich, MA). After digestion, DNA was ligated at room temperature using T4 DNA ligase (Promega). PCR conditions are shown in Table 3. Ligated products were confirmed by agarose gel electrophoresis and transformed using DH5α-competent cells. Positive clones were selected, subjected to plasmid isolation and purification using the Miniprep Kit, and verified by PCR and direct DNA sequencing.

**Table 3:** **PCR conditions used for DNA ligation for the construction of the human** ***IGF-IR*** **gene promoter (∞: hold)**

**Temperature**	**Time**
22°C	30 min
20°C	30 min
18°C	30 min
16°C	30 min
14°C	30 min
12°C	30 min
10°C	30 min
8°C	30 min
6°C	30 min
4°C	30 min
4°C	∞

### Site-directed mutagenesis and luciferase assay

Mutated human *IGF-IR* luciferase reporter constructs were generated using the QuickChange II XL Site Directed Mutagenesis Kit (Agilent Technologies, Santa Clara, CA) and a set of primers depicted in Table 4.

**Table 4:** **Sequences of the primers used to construct the 3 mutated fragments of human** ***IGF-IR*** **luciferase reporter (F1, F2, and F3)**

**Primer name**	**Sequence**
**F1** **(Forward)**	5′-CAA GAG CCC CAG CCG GGA GAA AGG GGA C-3′
**F1** **(Reverse)**	5′-GTC CCC TTT CTC CCG GCT GGG GCT CTT G-3′
**F2** **(Forward)**	5′-CAG AAA CGC GGA GCG CCG GCC ACC-3′
**F2** **(Reverse)**	5′-GGT GGC CGG CGC TCC GCG TTT CTG-3′
**F3** **(Forward)**	5′-GCC AGA GCG AGA GCG CCA AAT CCA GGA CAC-3′
**F3** **(Reverse)**	5′-GTG TCC TGG ATT TGG CGC TCT CGC TCT GGC-3′

Luciferase assay was performed with the Dual Glo Luciferase Kit (Promega) after co-transfecting the R^−^ cells with the reporter plasmids containing the wild-type or mutated *IGF-IR* promoter fragments or *NPM* promoter (kind gift from Dr. Qishen Pang, Cincinnati Children’s Hospital, Cincinnati, OH) along with Ik-1 or MZF1 expression plasmids using Lipofectamine 2000 (Invitrogen) for 48 h. Cells were trypsinized, washed twice with sterile PBS, and plated in a 96-well luminometer plate. An equal volume of Dual-Glo reagent was added and incubated for 10 min for cell lysis to occur. Firefly luminescence readings were obtained using a plate reader (PolarStar Omega, BMGLabTech, Cary, NC). Finally, the Dual-Glo Stop & Glo reagent was added and incubated for 10 min. Renilla luminescence readings were obtained using the above methods.

### Chromatin immunoprecipitation (ChIP) assay

Because NPM-ALK^+^ T-cell lymphoma cells contain low levels of endogenous Ik-1 and MZF1, expression plasmids containing full-length Ik-1 or MZF1 were constructed by transferring the inserts into a c-Myc-tagged expression vector (pCMV-Myc-N; Clontech). Briefly, the original expression vectors containing Ik-1 or MZF1 as well as pCMV-Myc-N were subjected to digestion using the restriction enzymes NotI/SalI for Ik-1 or XhoI/ECORI for MZF1 (Promega). Digested products were verified by agarose gel electrophoresis and then excised and ligated using the HD InFusion system (Clontech). Ligated products were transformed using DH5α-competent cells, and positive clones were selected and verified by PCR.

ChIP assays were performed using the Pierce Agarose ChIP Kit (Thermo Scientific). Briefly, at 48 h post-transfection, cells were cross-linked using 1% formaldehyde, and cell pellets were lysed and re-suspended in a buffer containing 0.6 μL Micrococcal Nuclease (ChIP grade) and subjected to sonication on ice (Output 6; six 15 sec pulses, followed by 45 sec rest periods; Sonic Dismembrator, model 100; Thermo Fisher Scientific). Five microliters of digested chromatin was separated for the 10% input. The remaining sonicated samples were immunoprecipitated overnight at 4°C on a rocking platform using the c-Myc-Tag antibody and the provided plugged spin columns. Following overnight incubation, ChIP-grade Protein A/G Plus agarose beads were incubated for 2 h with the lysate at 4°C on a rocking platform. The samples were then washed and reverse cross-linked at 65°C for 40 min. The immunoprecipitated samples and input were eluted in a buffer containing 5 M NaCl and 20 mg/mL Proteinase K. Finally, chromatin DNA was recovered and purified using the DNA Clean-Up column and subjected to Touchdown PCR using HotStarTaq Master Mix and Q solution and a set of primers shown in Table 5. PCR products were run on 1.5% agarose gel.

**Table 5:** **Sequences of the primers flanking potential binding sites (BS) of Ik-1 and MZF1 within the human** ***IGF-IR*** **promoter used in the RT-PCR reactions following the ChIP assay**

**Primer Name**	**Sequence**
**Ik-1 BS2** ** (Forward)**	5′-CGG GGG CAT TGT TTT TGG AG-3′
**Ik-1 BS2** ** (Reverse)**	5′-CGG GTT CCC CAA GAC GTG-3′
**Ik-1 BS3 (** **Forward)**	5′-TCT TGT TTA CCA GC ATTA ACT CGC-3′
**Ik-1 BS3** **(Reverse)**	5′-CCT CTC TCG AGT TCG CCT G-3′
**Ik-1 BS4** **(Forward)**	5′-CGC CGC TTT GTG TGT GTC-3′
**Ik-1 BS4** **(Reverse)**	5′-GCC GCC TCC TCC CTC A-3′
**MZF1 BS2** **(Forward)**	5′-GCG GGG GCA TTG TTT TTG GA-3′
**MZF1 BS2** **(Reverse)**	5′-CCG GGT TCC CCA AGA CGT G-3′
**MZF1 BS3** **(Forward)**	5′-GCG CGT GTC TCT GTG TGC-3′
**MZF1 BS3** **(Reverse)**	5′-GCG AGT TAA TGC TGG TAA ACA A-3′
**MZF1 BS4** **(Forward)**	5′-GTG TGT GTC CTG GAT TTG GGA-3′
**MZF1 BS4** **(Reverse)**	5′-GCA GAA ACG CGG AGT CAA AAT-3′
**MZF1 BS5** ** (Forward)**	5′-CGG CCC TTC GGA GTA TTG T-3′
**MZF1 BS5** **(Reverse)**	5′-CAA GTC TCA AAC TCA GTC TTC G-3′

### Western blotting

Cells were lysed using lysis buffer containing 25 mM HEPES (pH 7.7), 400 mM NaCl, 1.5 mM MgCl_2_, 2 mM EDTA, 0.5% Triton X-100, 0.1 mM PMSF, 2 mM DTT, and phosphatase and protease inhibitor cocktails (Thermo Scientific, Rockford, IL). Protein concentrations were measured using the Bio-Rad protein assay, and optical density values were obtained using an ELISA plate reader (Bio-Tek Instruments, Winooski, VT). Proteins (50 μg) were resolved by electrophoresis on 8% SDS-PAGE and then transferred to PVDF membranes and probed with specific primary antibodies and then with appropriate horseradish peroxidase-conjugated secondary antibodies (GE Healthcare, Cardiff, UK). Proteins were detected using a chemiluminescence-based kit (Amersham Life Sciences, Arlington Heights, IL).

In addition, a commercially available kit (Qproteome Tissue Kit, Qiagen, Valencia, CA) was used to perform Western blot assay to measure Ik-1 and MZF1 protein levels in formalin-fixed and paraffin-embedded tissue sections from NPM-ALK^+^ T-cell lymphoma patients (experiments performed on archived human tissues were in accordance with the Helsinki Declaration of 1975, as revised in 1983, and approval of the Institutional Review Board was obtained prior to performing such experiments). Briefly, tissue sections mounted on glass slides were examined and tumor areas were identified and marked. Next, sections were deparaffinized, and tumor areas were excised from the slides and transferred into 1.5-mL collection tubes. β-Mercaptoethanol (6 μL) was admixed with the provided Extraction Buffer EXB Plus (94 μL) and then added to the excised tissues. Tissue tubes were incubated on ice for 5 min and then the contents were mixed by vortexing and incubated on a heating block at 100°C for 20 min. Using an oven with rotators, samples were incubated at 80°C for 2 h with agitation at 750 rpm. After incubation, tubes were cooled at 4°C for 1 min. The samples were centrifuged for 15 min at 14,000 *g* at 4°C. The supernatant containing the extracted proteins was collected. For quantification of protein yield, the Bio-Rad assay was used as described above.

### MTS assay

Cell viability was evaluated using a CellTiter 96 AQueous One Solution Cell Proliferation 3-(4,5-dimethylthiazol-2-yl)-5-(3-carboxymethoxyphenyl)-2-(4-sulfophenyl)-2H-tetrazolium (MTS) assay kit (Promega). Cells were seeded in 96-well plates (1.0 × 10^4^ cells/well) in 100 μL RPMI supplemented with 10% FBS. Twenty microliters of MTS reagent were added, and the cells were incubated at 37°C in a humidified 5% CO_2_ chamber for 4 h. Optical density measurements were obtained at 490 nm using an ELISA plate reader.

### BrdU assay

Cell proliferation was measured using a BrdU assay kit (ExAlpha Biologicals, Shirley, MD). Briefly, 2.0 × 10^5^ cells/mL were plated into a 96-well plate. BrdU (1:500 dilution) was added, and the plate was incubated for 24 h at 37°C. Cells were then fixed for 30 min at room temperature. After the cells were washed, anti-BrdU antibody was added for 1 h followed by peroxidase goat anti-mouse IgG conjugate (1:2000 dilution) for 30 min. Next, the 3,3′,5,5′-tetramethylbenzidine peroxidase substrate was added, followed by incubation for 30 min at room temperature in the dark. The acid Stop Solution was then added and the plate was read at 450 nm using an ELISA plate reader.

### Cell migration assay

Cell migration was analyzed using 24-well Transwell plates with polycarbonate membranes (Corning Costar). Briefly, cells transfected with Ik-1 or MZF1 in serum-free culture medium were loaded into the upper compartment, and 500 ng/mL IGF-I (R&D Systems, Minneapolis, MN) in serum-free medium was loaded into the lower compartment. As controls, non-transfected cells in serum-free medium were loaded into the upper compartment with/without IGF-I loaded into the lower compartment. Plates were incubated for 4 h at 37°C, and cells migrating through the membrane into the lower chamber were counted using a particle counter and size analyzer (Beckman Coulter, Brea, CA).

### Anchorage-independent colony formation assay

Methylcellulose (Methocult H4230; StemCell Technologies) (3.0 mL) was added to 15-mL tubes. Empty vector- (EV-), Ik-1- or MZF1-transfected cells were added in a 1:10 (v/v) ratio to the methylcellulose tubes and mixed well by gentle inversion. One milliliter of the mix was divided into 24-well plates in triplicate. Plates were placed in a humidified incubator at 37°C in 5% CO_2_ for 7 days. Then, *p*-iodonitrotetrazolium violet was added for 24 h for staining. Colonies were visualized using the AlphaImager system (Alpha Innotech Corporation, San Leandro, CA). In an additional experiment, SUP-M2 cells were incubated for 21 days, and the results were similar to those of the shorter incubations.

### ***IGF-IR*** mRNA decay assay

Briefly, actinomycin D (Sigma) was dissolved in DMSO (final concentration: 1 mg/mL). Human T lymphocytes or NPM-ALK^+^ T-cell lymphoma cell lines were treated with 1 μM actinomycin D and samples were collected at 0, 0.5, 1, 2, 4, and 8 h. Total RNA was isolated and purified, and cDNA synthesis was performed as described above. Absolute real-time qPCR was used to measure the levels of *IGF-IR* mRNA in a 25-μL reaction by using 1 μL of the reverse-transcribed cDNA, 20× *IGF-IR* Taqman gene expression assay primer/probe, and 2× Universal PCR Mastermix (Applied Biosystems). To create a standard curve, serial 10-fold dilutions (30, 300, 3000, 30,000, and 300,000 copies) of an *IGF-IR* plasmid were used [36].

### Fluorescent in situ hybridization (FISH) assay to determine ***IGF-IR*** gene copy number

Human T lymphocytes and NPM-ALK^+^ T-cell lymphoma cells (10 × 10^4^ cells) suspended in RPMI were pipetted into cytospin chambers. Cytospin slides were prepared (700 rpm at high acceleration for 5 min). The cytospin slides were fixed in ice cold 100% methanol, and stored at −20°C until FISH was performed.

We adopted a previously described approach to perform FISH assay and analysis [72]. The SureFish probes and kit from Agilent Technologies were used. Briefly, 1.0 μL of *IGF-IR* FISH probe (G100168R) and 1.0 μL of chromosome enumeration probe 15 (CEP15; G100543G), which identify centromere 15, were mixed gently in Agilent FISH hybridization buffer. Cytospin slides were prepared and placed in gradually increasing concentrations of ethanol (70%, 85%, and 100%), each for 1 min at room temperature. After allowing the slides to dry, 5.0 μL of probe/hybridization buffer mixture were added to the slides, and cover slips were applied. Hybridization was then accomplished by using the ThermoBrite system (Abbott Molecular, Abbott Park, IL). The slides were first incubated at 78°C for 5 min to denature the DNA, and then incubated at 37°C for 24 h. Thereafter, cover slips were removed and slides were placed and agitated in Wash buffer 1 (73°C) for 2 min. Subsequently, slides were transferred and agitated at room temperature for 2 min in Wash Buffer 2. The slides were air dried in the dark at room temperature, followed by pipetting DAPI (1.0 μg/mL in PBS). DAPI was then removed, and slides were mounted with ProlongGold antifade media (P36934, Invitrogen, Grand Island, NY) and viewed using the FV1000 confocal microscope (Olympus America, Center Valley, PA).

FISH scoring was performed in 55 nonoverlapping nuclei per slide. The means of the *IGF1R* gene and CEP15 copy numbers per cell, number of cells with two or fewer, three, and four or more copies of *IGF1R* and CEP15 signals, and *IGF1R*-to-CEP15 ratio were obtained.

### Measurement of IGF-I levels secreted by NPM-ALK^+^ T-cell lymphoma cells

After transfection of scrambled or ALK siRNA for 48 h, cells were transferred to serum free medium for 24 h. The medium was then collected and concentrated using Amicon Ultra-15 centrifugal tubes (UFC900308; Millipore). ELISA assay was performed using the IGF-I cytokine kit (R&D Systems).

### Statistical analysis

Statistical analysis was performed using the PRISM software (GraphPad, La Jolla, CA). Statistical significance was detected using one-way ANOVA and Bonferroni’s post hoc multiple comparisons test. *P* < 0.05 was considered statistically significant.

## Additional file

Additional file 1:
**Supporting Results.**
