# Supplementary material for: The transcription factors Ik-1 and MZF1 downregulate IGF-IR expression in NPM-ALK+ T-cell lymphoma
Source: Mol Cancer. 2015 Feb 25;14:53. doi: 10.1186/s12943-015-0324-2 (PMC4415347; doi:10.1186/s12943-015-0324-2)
Supplement: Additional file 1: — Supporting Results. [file 12943_2015_324_MOESM1_ESM.pdf]

SUPPORTING RESULTS

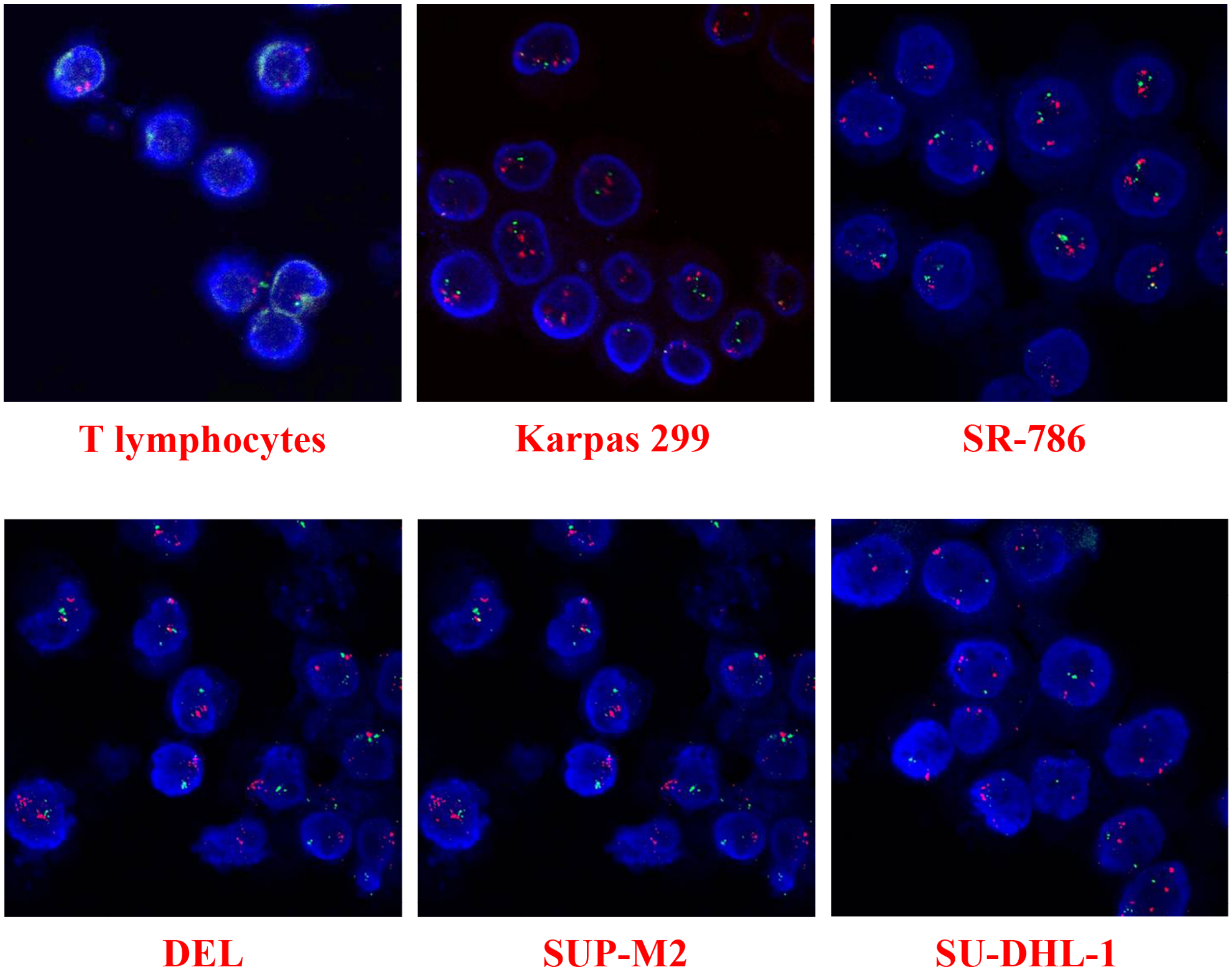

Figure 1-AF. FISH analysis of *IGF-IR* gene (red signal) and chromosome enumeration 15 (green signal; CEP15), which detects centromere 15, copy numbers in normal human T lymphocytes and NPM-ALK$^+$ T-cell lymphoma cell lines.

FISH studies show no evidence to support amplification of *IGF-IR* gene in NPM-ALK$^+$ T-cell lymphoma cells. As shown in Table 1-AF, the *IGF-IR*-to-CEP15 ratio was consistently < 2.0 in the T lymphocytes as well as in the NPM-ALK$^+$ T-cell lymphoma cells.

Table 1-AF. The average number of *IGF-IR* and CEP15 copies per cell as detected by FISH analysis.

	IGF-IR	**CEP15**	***IGF-IR*-to-CEP15 ratio**
**T lymphocytes**	1.51	1.53	0.99
**Karpas 299**	3.02	2.56	1.18
**SR-786**	4.18	3.89	1.07
**DEL**	4.44	4.44	1.00
**SUP-M2**	2.54	1.94	1.31
**SU-DHL-1**	2.65	2.13	1.24
